# Predicting methicillin-resistant *Staphylococcus aureus *in critically ill patients with pneumonia presenting to the hospital

**DOI:** 10.1186/cc10650

**Published:** 2012-03-20

**Authors:** AF Shorr, DE Myers, DB Huang, BH Nathanson, MF Emmons

**Affiliations:** 1 Washington Hospital Centre, Washington, DC, USA; 2 Pfi zer, Inc., Collegeville, PA, USA; 3 VA NJ Healthcare System, East Orange, NJ, USA; 4 OptiStatim, LLC, Longmeadow, MA, USA; 5 Cerner LifeSciences, Beverly Hills, CA, USA

## Introduction

Methicillin-resistant *Staphylococcus aureus *(MRSA) represents an important pathogen in those presenting to the hospital with pneumonia and requiring ICU admission. However, empiric treatment against MRSA in those admitted to the ICU with severe non-nosocomial pneumonia could lead to overuse of anti-MRSA therapy. To address this concern, we sought to develop a simple clinical score for identifying ICU patients presenting to the hospital with pneumonia unlikely to be caused by MRSA.

## Methods

We retrospectively identified patients admitted to the ICU with community-acquired pneumonia (CAP) or healthcare-associated pneumonia (HCAP) between April 2007 and March 2009 at 62 hospitals in the USA. The diagnosis of pneumonia was based on ICD-9 codes. We only included patients with laboratory evidence of bacterial infection (for example, positive sputum, blood, pleural cultures or urinary antigen testing). We determined, via logistic regression, variables independently associated with the presence of MRSA (two-thirds of cohort) and developed a risk score based on this. We then internally validated (one-third of cohort) the score.

## Results

The cohort included 957 patients (mean age 65.8 ± 16.4 years, 50.2% male, 43.7% HCAP). MRSA was identified in 20.1%. The risk score assigned points as follows: 1 point - age <30 or >79 years, recent immunosuppression other than corticosteroids, shock; 2 points - admission from a skilled nursing facility, history of diabetes without coronary artery disease (CAD) or heart failure without CAD. The prevalence of MRSA increased with escalating score (*P *< 0.001). We collapsed the score into three strata based on risk for MRSA (score of 0 to 1 (low), 2 to 4 (moderate), ≥5 (high)). The respective MRSA rates by strata equaled 15.2%, 24.7%, and 31.9%, (*P *< 0.001). A score ≤1 as a screening test to exclude MRSA performed poorly (sensitivity 58.3%, specificity of 53.3%).

## Conclusion

The prevalence of MRSA in patients with CAP or HCAP requiring ICU care was high. A score to assess the risk for MRSA in these patients performed poorly but requires external validation. Given the high risk of MRSA in this setting along with the limited discriminatory power of our risk score, empiric therapy for MRSA in these patients seems appropriate.

